# Diversity and taxonomy of giant algal viruses: chloroviruses lead the way

**DOI:** 10.1128/jvi.01688-25

**Published:** 2026-05-14

**Authors:** Rodrigo A. L. Rodrigues

**Affiliations:** 1 Virus Laboratory, Department of Microbiology, Federal University of Minas Gerais 28114, Belo Horizonte, Minas Gerais, Brazil; Universiteit Gent, Merelbeke, Belgium

**Keywords:** giant virus, phycodnavirus, chlorovirus, *Algavirales*, algal virus, virus taxonomy

## Abstract

Viruses are the most diverse and abundant biological entities on Earth. Their impact on life is undeniable, and viruses of microorganisms, in particular, exert a profound impact on the planet by shaping biogeochemical cycles and driving the evolution of living organisms. Among them, the giant viruses of the family *Phycodnaviridae* are major players in aquatic environments, infecting many groups of algae from inland and marine ecosystems. The discovery of chloroviruses over 40 years ago started a new era in virology, paving the way for fundamental discoveries into the diversity and evolution of giant viruses, and ultimately leading to the establishment of the order *Algavirales,* included in the phylum *Nucleocytoviricota*. In recent years, dozens of chlorovirus genomes have been sequenced, greatly expanding our understanding of the extent of their diversity and evolution. This review provides an overview of the diversity of giant algal viruses of the order *Algavirales* and compiles the most recent data on the genomic landscape of chloroviruses based on 130 sequenced viruses. Genetics reflects biological diversity, as evidenced by the distinct plaque morphotypes, which range from tiny (<1 mm) and diffuse plaques to very large (>4 mm) and well-defined plaques. Such diversity is likely to be present in other algal viruses, and the mechanisms behind this remain to be evaluated. Moreover, challenges related to the taxonomy of phycodnaviruses are discussed, and a new perspective for *Algavirales* is presented based on comparative genomics and phylogenetics, suggesting the existence of new virus species yet to be discovered.

## INTRODUCTION

How vast and diverse is the virosphere? This fundamental question, often regarded as mere scientific curiosity, has been central to the advancement of virology since its beginning in the late 19th century. Several studies suggest the existence of numerous viruses on Earth, with estimates on the order of 10^31^ viral particles worldwide (1, 2). Clearly, this does not imply the existence of an equivalent number of viral species. Nevertheless, a vast number of viral species remain to be discovered, and efforts aimed at viral classification may provide valuable insights into this foundational question. According to the Natural History Museum in London, approximately 2.1 million species of living organisms had been cataloged by 2024, excluding microorganisms (3). Estimates suggest that there should be around 1 trillion (10^12^) microbial species on our planet (4). If each living species were associated with a single viral species, one could conservatively estimate the existence of more than one trillion viral species. However, according to the most recent update from the International Committee on Taxonomy of Viruses (ICTV; August 2025, https://ictv.global/taxonomy), we know of just around 17,500 viral species. There is still a long way to go before we have a minimally accurate answer to that fundamental question.

This immense diversity is intrinsically linked to the profound impact viruses exert on the biosphere and the organisms that inhabit it. Among their most significant ecological roles are their functions as biological control agents and key drivers of the biogeochemical cycle. In aquatic environments, viruses, and particularly those infecting microorganisms, play a major role in carbon cycling by lysing host cells and converting biomass into particulate organic carbon and dissolved organic carbon, thereby facilitating the transfer of organic matter to higher trophic levels (5–10). Viruses of microorganisms have been known for more than a century, since bacteriophages were discovered in the early 20th century by Félix d’Herélle and Frederick Twort. However, it was not until the second half of the 20th century that viruses associated with phytoplankton were first described. These initial discoveries involved cyanophages infecting *Microcystis aeruginosa* and marked a paradigm shift in virology, expanding the field’s scope and paving the way for the discovery of a remarkable diversity of viruses associated with algae (11, 12).

Algae are fundamental components of phytoplankton. This is a broad, non-taxonomic term that often encompasses prokaryotes (e.g., cyanobacteria) and eukaryotes, but for the purposes of this review, it will always be used to refer to eukaryotic algae. According to AlgaeBase, approximately 62,000 algal species had been described as of December 2025 (13). Viruses have already been described as being associated with some species of algae, including both RNA and DNA viruses (12, 14–18). However, as with the entire virosphere, only a small fraction of this diversity is currently known. Among the characterized algal viruses, the most extensively studied and structurally complex are the giant DNA viruses of the order *Algavirales*. This minireview focuses on these viruses, with particular emphasis on chloroviruses, which were first identified in the late 1970s and revealed an unprecedented level of complexity in virology (14, 19). These discoveries ultimately led to the identification and characterization of several giant viruses that are now classified within the phylum *Nucleocytoviricota* (20, 21). Here, I compile the most recent data on chlorovirus diversity, which has recently undergone a taxonomic update by the ICTV. I also highlight how this represents an important first step toward the reclassification of other giant algal viruses. Using comparative genomics, I provide new information on the diversity of these viruses and aim to establish a solid framework for understanding the current taxonomic challenges, how to address them, and what we can expect in the coming years.

## CHLOROVIRUSES, THE FIRST GIANT ALGAL VIRUSES

The first report of a large virus associated with an alga was in the late 1970s, when icosahedral particles measuring up to 180 nm in diameter were visualized inside the protozoan *Paramecium bursaria* (19). These particles were associated with endosymbiotic algae of the genus *Chlorella*, commonly referred to as zoochlorella, and the virus was subsequently named Zoochlorella virus (ZCV). The virus was capable of replicating within the algal host but not within the paramecium itself, representing the first description of a complex, multi-organism interaction involving a large algal virus. A few years later, in 1981, a new virus was isolated from a Chlorella associated with the coelenterate *Hydra viridis* and was named Hydra viridis Chlorella virus (HVCV) (22). This virus exhibited large, electron-dense icosahedral particles that replicated within the algal cytoplasm and possessed a large double-stranded DNA (dsDNA) genome. Shortly after, James Van Etten and colleagues reported two other new large viruses associated with Chlorella, found in both *Hydra viridis* and *Paramecium bursaria* zoochlorella, the latter named Paramecium bursaria Chlorella virus 1 (PBCV-1) (23, 24), which would become the model for studying large algal viruses and be responsible for several advances in the field, as will be demonstrated in this minireview.

In 1990, the ICTV approved the creation of the family *Phycodnaviridae* to classify polyhedral dsDNA viruses that infect algae of the genus *Chlorella*, designating PBCV-1 as the type species of the genus Phycodnavirus, which was later renamed *Chlorovirus* (25). In this context, a chlorovirus can be defined as a large, icosahedral dsDNA virus that replicates in certain unicellular, Chlorella-like green algae and is widely distributed in freshwater environments worldwide (26, 27). Much of our current understanding of chloroviruses stems from the pioneering work of Professor James Van Etten (University of Nebraska-Lincoln, USA) and his collaborators, who have studied these viruses for more than 45 years, using PBCV-1 as an experimental model (14). In recognition of his contributions and in accordance with current viral taxonomy standards, the species *Paramecium bursaria* Chlorella virus 1 was renamed *Chlorovirus vanettense* in 2023, honoring the researcher who inaugurated the field of giant algal virus research.

**Fig 1:**
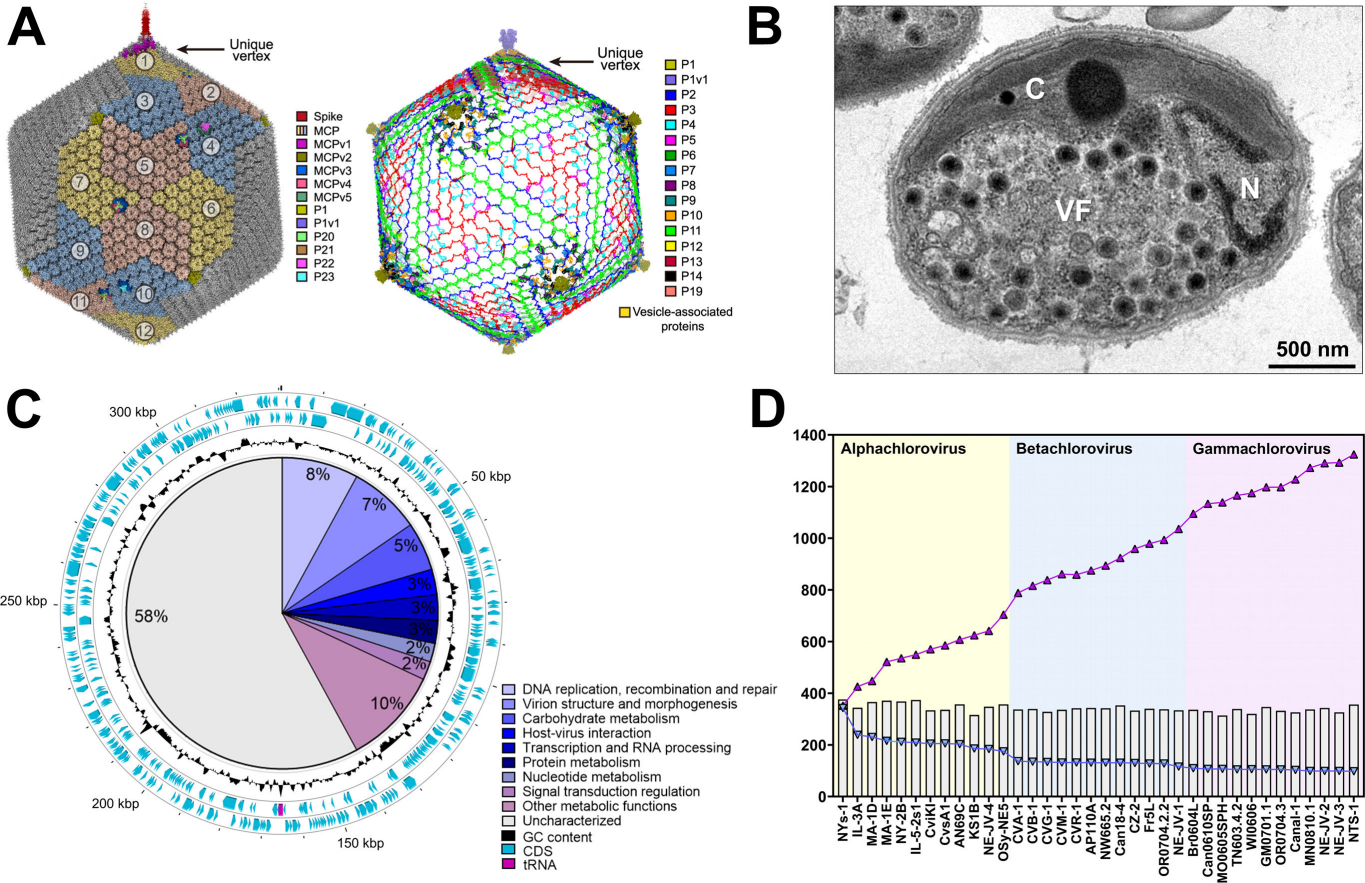
General features of PBCV-1 and other chloroviruses. (**A**) Structure of the PBCV-1 capsid; 5-fold averaged cryo-EM map of the viral capsid. Cryo-EM densities of the spike and capsid proteins are colored according to the color key. Numbers indicate the icosahedral asymmetric units within the 5-fold asymmetric unit. The unique vertex is indicated with a black arrow (reproduced from reference 29, previously published under a CC BY 4.0 license). (**B**) Transmission electron microscopy image of a *C. variabilis* NC64A alga cell infected with a chlorovirus isolated in Brazil at a late stage of infection. Viral particles occupy a large fraction of the cytoplasm, forming the viral factory (VF). Nucleus (N) and chloroplast (Ch) remain intact. The image was obtained from the Microscopy Center of UFMG using a Tecnai G2-12 Spirit Biotwin at 120 kV and is part of the image collection of the Brazilian Study Group of Phycodnaviruses. (**C**) Genomic map and gene content of PBCV-1. Outer rings indicate the CDSs and tRNA, and the inner ring indicates the GC content. The circular representation of the linear genome was generated using Proksee (42). The pie chart shows functional gene annotation categorized based on NCVOG categories. Gene reannotation was performed, considering the 416 CDS predicted for PBCV-1 (41), using BLASTp searches against the ClusteredNR (nr_cluster_seq) database with an e-value cutoff of 10^−5^ in December 2025. (**D**) Pan-genome evolution considering 36 chlorovirus isolates equally distributed among the three subgenera of *Chlorovirus*. Subgenera are indicated by different background colors. Bars represent the number of CDSs per isolate, the purple line with triangles indicates the pan-genome, and the blue line with squares indicates the core genome. The graph was generated using data from Rodrigues et al. (43) and plotted with GraphPad Prism 9.

Numerous studies have demonstrated the structural and biological complexity of chloroviruses. Based on PBCV-1, near-atomic resolution structural biology studies have revealed a complex 190 nm particle with pseudoicosahedral symmetry (T = 169 d). The particle consists of two protein layers: an outer layer composed of multiple proteins, dominated by the major capsid protein Vp54 (gene *a430l*), with 4,905 copies, and an inner layer formed by at least 18 minor capsid proteins (mCPs; P2–P19), which provide the structural framework of the virion, along with additional proteins (P20–P23) that form short fibers attached to the capsid (28, 29) (Fig. 1A). The MCP and its variants (at least five variants have been described to date) have different amounts of attached glycans, with glycans synthesized by virus-encoded enzymatic machinery rather than host pathways (29, 30). The virus has an internal lipid membrane that surrounds the viral genome, a characteristic common to several giant viruses of the phylum *Nucleocytoviricota,* and which appears to be a remnant of its ancestors (31, 32). In addition, a distinctive spike structure located at a unique vertex of the particle is believed to mediate host cell recognition and initiation of the infection process (33, 34) (Fig. 1A). The life cycle of chloroviruses is similar to that observed for some bacteriophages (34). Following attachment to the host cell, partial degradation of the algal cell wall occurs, allowing delivery of the viral genome into the cytoplasm (35, 36). The PBCV-1 DNA is packaged under pressure, which aids in getting the DNA into the cell (37). The reduction in the host turgor pressure by the depolarization of the host membrane also contributes to this effort to get the DNA into the cell. Viral DNA replication and transcription take place in the nucleus within the first hour post-infection (hpi) with at least 50 PBCV-1 genes being expressed by 7 min post-infection (38), whereas virion assembly occurs in the cytoplasm with subsequent cell lysis and release of viral progeny within 8 hpi, in the case of PBCV-1 (39, 40). Both nuclear and chloroplast DNA are degraded shortly after the virus infection. During morphogenesis, it is possible to observe numerous viral particles in different degrees of maturation occupying most of the host cytoplasm, with little or no alteration in the nucleus and chloroplast (Fig. 1B). Proteomic analyses indicated the presence of 148 proteins composing the viral particle after release from the cell, 147 of which are encoded by the viral genome (41).

Sequencing and analysis of the PBCV-1 genome revealed a large linear dsDNA molecule, making the first viral genome to exceed the 300 kbp barrier and remaining the largest known viral genome until the end of the 20th century (44). The most recent GenBank entry (accession no. JF411744) reports a PBCV-1 genome of 330,611 bp in size, with a GC content of 40% and hundreds of open reading frames (ORFs), of which 416 were considered coding sequences (CDSs) with at least 40 codons in length and without major overlaps and showing evidence of transcription during infection and/or incorporation into virions, as well as 11 tRNA genes (41). The CDSs are evenly distributed across both DNA strands, whereas the tRNA genes are clustered near the center of the genome (Fig. 1C). Functional annotation of PBCV-1 genes revealed a large representation of proteins involved in DNA replication and repair (8%), virion structure and morphogenesis (7%), and carbohydrate metabolism (5%), including an unusually sophisticated viral glycosylation system, something unusual in the virosphere but may be reasonably common among giant viruses (45). In contrast to many other giant viruses, PBCV-1 has limited transcriptional machinery (3%), including some transcription factors but lacking RNA polymerase, making this virus dependent on functional host gene transcription (Fig. 1C). The isolation and genomic characterization of other chloroviruses, as well as the discovery of viruses with large genomes infecting other algal species, consolidated these as the first giant viruses (46). These findings laid the foundation for one of the most significant paradigm shifts in modern virology: the discovery and characterization of viruses with giant particles (>1,500 nm) and exceptionally large genomes (>2.5 Mbp) that infect protists, thereby blurring the original concept of viruses established in 1957 by André Lwoff (47). For further details, the readers are referred to recent reviews on giant viruses (20, 48, 49).

## EXPANDING THE DIVERSITY OF CHLOROVIRUSES AND THE PATH TOWARD A NEW TAXONOMY OF GIANT ALGAL VIRUSES

Following the isolation of PBCV-1, several other chloroviruses were isolated, reinforcing the ubiquitous nature of these viruses in inland aquatic environments. Chloroviruses have been isolated from different regions worldwide, but mostly in the northern hemisphere, with several isolates originating from North America, Europe, and some from Asia (50). It is still uncertain which are the natural hosts of chloroviruses. To date, there are reports of chloroviruses being isolated from three species of algae, all endosymbionts of protists or animals. Several viruses, including PBCV-1, have been isolated from the *C. variabilis* strain NC64A and were historically designated NC64A-viruses. Other viruses have been isolated from *Chlorella heliozoae* strain SAG3.83, an alga that establishes endosymbiosis with the heliozoan *Acanthocystis turfacea*, with Acanthocystis turfacea Chlorella virus 1 (ATCV-1) being the first to be isolated and characterized from a water sample from a university in Germany (51, 52). This and other viruses isolated from the same host were named SAG-viruses. Furthermore, some viruses were isolated from *Micractinium conductrix* strain Pbi, previously designated *Chlorella* Pbi, an endosymbiont of *Paramecium bursaria* that was originally isolated in Europe (53, 54). These viruses were called Pbi-viruses. Although chloroviruses exhibit several similarities, such as size and genetic content, and infect algae that are relatively close to one another from an evolutionary standpoint, they are very specific to their hosts. To date, no chlorovirus has been shown to infect more than one algal species. In 2017, a novel chlorovirus capable of infecting *Chlorella variabilis* strain Syngen 2–3 was isolated and characterized. Notably, although this virus was highly similar to other NC64A viruses and clustered phylogenetically within the NC64A clade, it replicated exclusively in the Syngen 2–3 lineage, resulting in the definition of OSy-virus or Only Syngen virus (55). Subsequently, it was observed that viruses isolated from the *C. variabilis* strain NC64A were able to infect the Syngen 2–3 lineage, but the reverse was not true. Although some of the OSy-viruses can enter the NC64A cells, genome replication does not occur, resulting in abortive infection (56).

**Fig 2:**
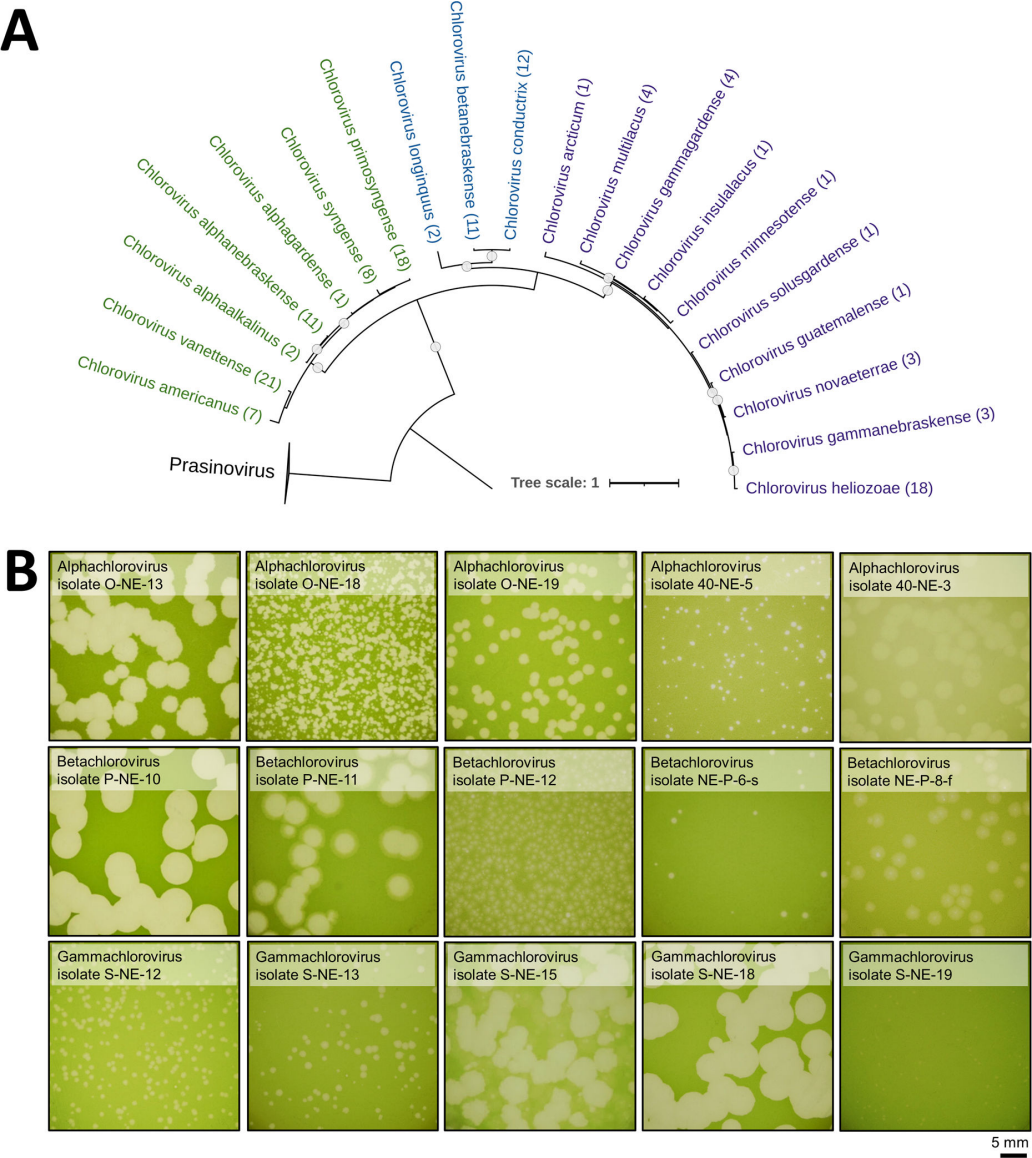
Phylogenetic and biological diversity of chloroviruses. (**A**) Phylogenetic tree indicating the proposed 20 species of the genus *Chlorovirus*. The tree was drawn based on the data from Rodrigues et al., 2025 (60), using iToL v6 (61). Prasinoviruses were used as the outgroup, and the scale bar represents the amino acid substitution rate. Alphachloroviruses are shown in green, betachloroviruses in blue, and gammachloroviruses in purple. Numbers in parentheses indicate the number of virus isolates within each species. (**B**) Plaque morphology variation among chloroviruses. Different isolates, even from the same subgenera, can exhibit very different plaque phenotypes, with plaque sizes ranging from < 1 to >4 mm in diameter. All infections were performed using plaque-purified viruses; nevertheless, it is not uncommon to observe more than one plaque morphology even after multiple rounds of plaque purification, as observed for Alphachlorovirus isolate O-NE-18 and Gammachlorovirus isolate S-NE-15. Some isolates form regular plaques with cloudy edges (Betachlorovirus isolate P-NE-11). Some isolates form very small and faint plaques (Gammachlorovirus isolate S-NE-19), while others form very large and regular plaques (Gammachlorovirus S-NE-18). All pictures are in the same scale to allow proper comparison of the plaque sizes. Scale bar: 5 mm. Images courtesy of Roger Carlson.

Based on the analysis of 36 chlorovirus genomes encompassing all groups described at the time, we described an open pan-genome comprising 1,345 clusters of orthologous groups of genes (COGs), of which only 126 were shared among all viruses, constituting the core genome (43) (Fig. 1D). Approximately two-thirds of the chlorovirus pan-genome lacked functional annotation, highlighting the need for additional studies involving virus isolation and detailed genomic and biological characterization. Such efforts are essential for improving our understanding of chlorovirus diversity and evolution. With this in mind, dozens of new isolated chloroviruses, many obtained from samples of alkaline lakes in the Sandhills region of Nebraska, USA, were sequenced and analyzed, revealing a previously unexplored diversity of chloroviruses (57–59). With a total of 68 viruses isolated from *C. variabilis*, including 30 OSy-viruses, it was possible to obtain a general genomic overview of these viruses and improve the understanding of their evolution, which triggered a taxonomic reclassification of chloroviruses (57). The genome of these viruses varies from 288 kbp to 410 kbp, encoding between 319 and 463 CDSs and containing between 6 and 18 tRNA genes. As expected, there is a direct proportionality relationship between genome size and the number of CDSs, but the same is not observed when considering genes that code for tRNA. As an example, the chlorovirus isolate O-NE-23 has the largest genome (409,489 bp), the highest number of CDSs (*n* = 463), but the fewest number of tRNAs (*n* = 6) among *C. variabilis*-infecting chloroviruses (57). With a larger amount of data about OSy-viruses, we observed that these viruses evolved from a subgroup of NC64A viruses. For reasons that remain unclear, some OSy-virus isolates appear to have expanded their host range and are capable of infecting *C. variabilis* strains other than Syngen 2–3 (57). These findings demonstrate that host-based classification, which has historically been used informally for chloroviruses, is not sustainable as a long-term taxonomic strategy. To address this limitation, we proposed designating viruses that infect and replicate in any tested *C. variabilis* strain as members of the subgenus “Alphachlorovirus” (57). Using comparative genomics analyses, we demonstrated that alphachloroviruses exhibit good conservation in genomic structure, but there is significant variation at the nucleotide sequence level. Based on average nucleotide identity (ANI) analyses, we discovered that a threshold of 94% identity corresponded well with phylogenetic clustering, leading to the proposal of seven alphachlorovirus species (57) (Fig. 2A). Despite the availability of extensive genomic data, the alphachlorovirus pan-genome remains open, indicating that additional virus isolation efforts will continue to expand the known genetic diversity of this group.

Shortly after the isolation of ATCV-1 in Germany, other SAG-viruses were identified in different regions of the planet, including the USA, Canada, and Brazil, among others, demonstrating the ubiquity of this group of chloroviruses (50). To date, a total of 37 SAG viruses have been isolated and sequenced, revealing an even more surprising diversity when compared to alphachloroviruses (58). These viruses have been designated Gammachloroviruses. Their genomes range from 283 kbp to 385 kbp, encode up to 420 CDSs, and contain up to 13 tRNA genes. Gammachloroviruses also exhibit a substantially higher GC content than alphachloroviruses, with an average of approximately 49%, compared to 40% in the latter group. Notably, one isolate (Chlorovirus MN0810.1) has a GC content of 52%, the highest reported among chloroviruses to date. ANI-based analyses revealed markedly greater genomic divergence among gammachloroviruses than among alphachloroviruses, leading to the definition of 10 species of gammachlorovirus (58). Some species are currently represented by a single isolate, such as Chlorovirus GNLD-22, the only giant virus isolated from Greenland to date, whereas others include multiple isolates, with up to 18 members, as in the case of *Chlorovirus heliozoae*, which includes ATCV-1 (58) (Fig. 2A). As with alphachloroviruses, gammachloroviruses exhibit relatively high conservation in genomic organization and an open pan-genome. Interestingly, viruses isolated from geographically and ecologically disparate locations (e.g., São Paulo, Brazil, and Tennessee, USA) can belong to the same species, sharing more than 94% genomic identity. Conversely, viruses isolated from the same lake in Nebraska may show substantial nucleotide divergence and belong to distinct species (58). This suggests an absence of clear ecological barriers as a determining factor for the diversity of chloroviruses. However, more studies should be carried out to test this hypothesis. One potential exception may be polar environments, as suggested for other giant viruses (62). It is noteworthy that the chlorovirus isolated from Greenland is considerably different from the others, emphasizing the importance of expanding virus isolation efforts in polar regions.

The third group of chloroviruses is the least diverse in genetic terms, although it is also the group with the fewest number of viruses isolated and sequenced to date (*n* = 25) (59). The Pbi-viruses, now called beta-chloroviruses, have been isolated exclusively in North America (USA and Canada) and in some European countries. These viruses replicate in the alga *Micractinium conductrix* Pbi and were the second group of chloroviruses to be isolated and characterized, with the first isolates obtained in the 1980s (54). Through phylogenetic and comparative genomic analyses, we showed that betachloroviruses are well conserved from a genomic point of view, currently comprising only three species (59). However, it is noteworthy that one of the species is very divergent from the other two, forming a long branch in phylogenetic analyses. Sequencing of Chlorovirus NE-JV-1 indicated a large divergence in relation to other chloroviruses, with low genetic similarity and large divergence in terms of genomic organization, having been considered a phylogenetic outlier for a long time (50). Although it is only capable of replicating in *M. conductrix* and shares high genetic similarity with other betachloroviruses, it is noteworthy that, depending on the phylogenetic marker used, especially if used in isolation, it commonly ends up closer to the gammachloroviruses (59). The recent isolation of Chlorovirus NE-P-6-s, which shares more than 98% nucleotide identity with NE-JV-1 and exhibits a similar genome structure, confirmed that these divergent viruses are present across different locations and highlighted the importance of further sampling to resolve phylogenomic gaps (59). An intriguing observation emerging from beta-chlorovirus studies is that isolates with extremely high genomic similarity (ANI >99%) can display markedly different plaque morphologies in culture, which led us to apply the term “genomovar,” or genomic variants, for chloroviruses (59). Chloroviruses can form large lysis plaques in cell culture (over 4 mm in diameter), although others form very small plaques (less than 1 mm) (63). This fascinating variation in plaque morphology, especially considering viruses with very high genomic similarity, is a phenomenon we have observed for all chlorovirus groups (Fig. 2B).

When only a small number of viruses are known, and their genomic and biological properties remain poorly characterized, our perception of viral diversity is inherently limited, constraining taxonomic progress. With a data set of 130 chlorovirus genomes, we changed this situation. Considering this number of sequenced viruses, we have predicted a total of 49,538 chlorovirus genes, with 26,041 genes in alphachloroviruses; 9,431 genes in betachloroviruses; and 14,066 genes in gammachloroviruses. This abundance of genetic data significantly improved our understanding of chlorovirus diversity and was a major driver for a taxonomic update. By considering multiple lines of evidence, including nucleotide similarity (as emphasized by ICTV species demarcation guidelines), host range, genome organization, and phylogenetic relationships, we proposed the establishment of three subgenera within *Chlorovirus* and delineated a total of 20 viral species (60) (Fig. 2A). We believe that this was the first step toward a broader update of the taxonomy of giant algal viruses and that the rationale and analyses developed can also be expanded to other members of the order *Algavirales*. In the following sections, I will address these ideas in more detail and provide an overview of what may happen in the near future for this historic group of giant algal viruses.

## *ALGAVIRALES*, AN ATTEMPT TO ORGANIZE THE GIANT ALGAL VIRUSES

**Fig 3:**
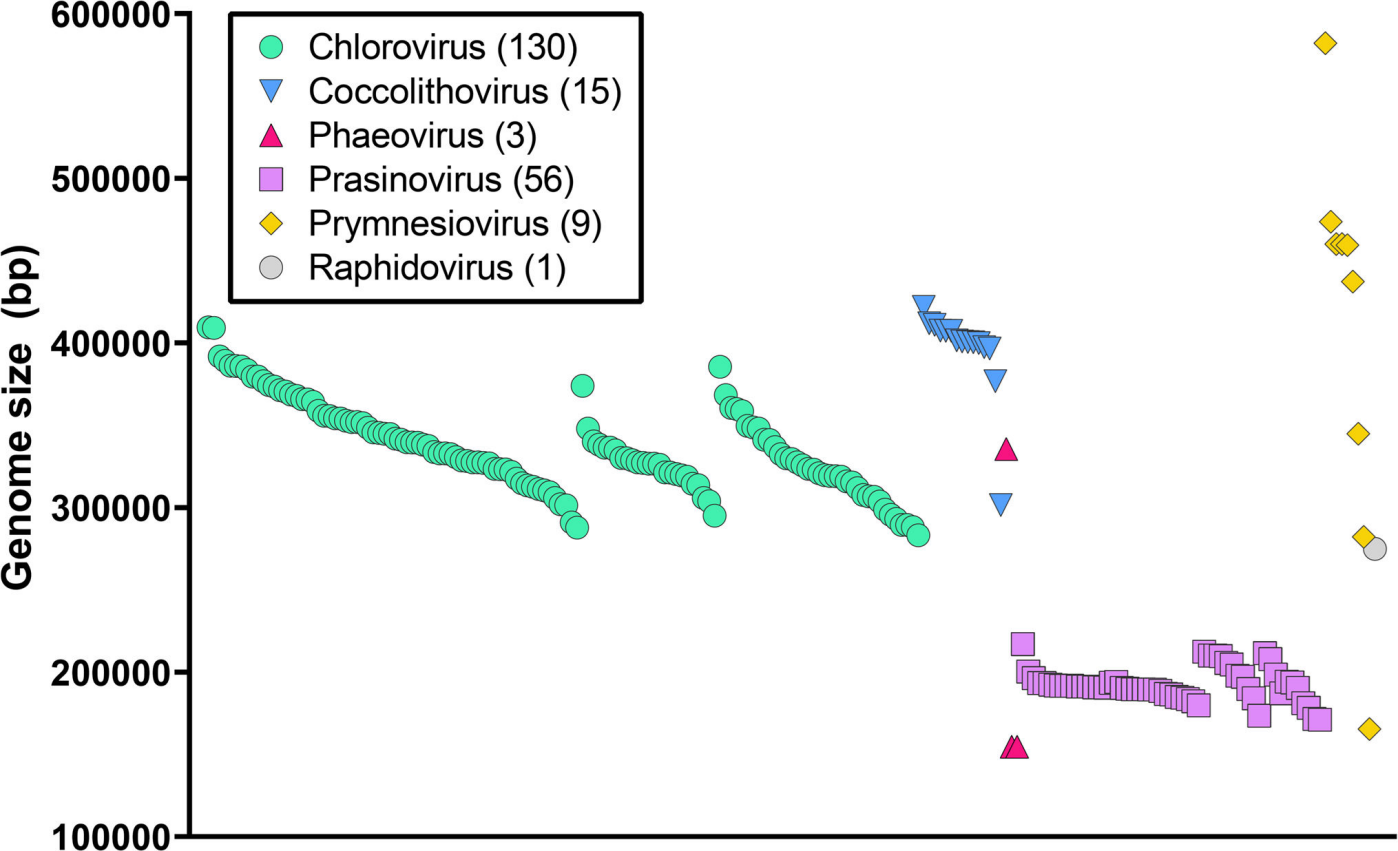
Genome sizes and distribution of *Algavirales*. Distribution plot of genomes of viruses belonging to the order *Algavirales*, considering the six genera currently recognized by the ICTV. Genera are represented by different symbols and colors, as indicated in the color key. All complete or near-complete genomes associated with the taxon “Algavirales” (taxid: 2732524) were retrieved from the GenBank by December 2025 (*n* = 214). The y-axis indicates the viral genome size. Numbers in parentheses indicate the number of genomes available for each genus within the family *Phycodnaviridae*.

The creation of the family *Phycodnaviridae* in 1990 opened doors for the classification of other algal DNA viruses. In 1998, the ICTV ratified the creation of three new genera within this family, grouping viruses associated with different lineages of marine algae. The genus *Prasinovirus* was initially created to classify viruses infecting *Micromonas* sp., a ubiquitous marine microalga. The first isolated virus, named Micromonas pusilla virus SP1, was considered the prototype of the genus and currently belongs to the species *Prasinovirus micromonas* (64, 65). A few years later, viruses associated with another species of marine algae, *Ostreococcus tauri*, were isolated and included in the group (66, 67). Although many *Ostreococcus* sp. viruses have been reported (68), only the species *Prasinovirus ostreotauri* is registered in the ICTV to date. In addition, a third group of viruses has been reported in recent years exhibiting phylogenetic proximity to prasinoviruses (69). Different isolates of *Bathycoccus* sp. virus have been characterized, but the overall diversity of this group remains poorly understood (70). After *Chlorovirus*, the genus *Prasinovirus* has the largest number of isolated giant algal viruses with sequenced genomes available in GenBank to date (Fig. 3).

The genus *Prymnesiovirus* was the third to be incorporated into the family *Phycodnaviridae*, with the Chrysochromulina brevifilum virus PW1 considered the prototype of the genus, currently belonging to the species *Prymnesiovirus brevifilum* (71). Additional prymnesiophyte-associated viruses have since been isolated, including viruses infecting *Phaeocystis globosa* and *Chrysochromulina parva*, which maintain phylogenetic affinity with other phycodnaviruses (72, 73). Notably, some viruses isolated from these same algal hosts, such as Chrysochromulina parva virus BQ1, and some *Phaeocystis globosa* viruses are highly divergent and cluster phylogenetically closer to members of the family *Mimiviridae*, whose members predominantly infect heterotrophic protists (73–76). It is noteworthy that 20 years ago, two groups of *Phaeocystis globosa* viruses were discovered, differing in particle size and genome length (77). These viruses were later shown to belong to two separate viral families, underscoring the remarkable diversity of algal viruses and highlighting how much remains to be discovered (78). Also in 1998, a fourth group of phycodnaviruses named *Phaeovirus* was established to classify viruses associated with multicellular algae of the class Phaeophyceae, with Ectocarpus siliculosis virus 1 (EsV-1) considered the prototype of the genus at the time (79). Currently, there are only three species of phaeoviruses associated with algae of the genera *Ectocarpus* and *Feldmannia*. These are the phycodnaviruses with the smallest genomes described to date, at approximately 160 kbp (Fig. 3). Unlike other phycodnaviruses, phaeoviruses have a latent infection cycle, integrating their genomes into the host DNA, as observed in viruses infecting kelp (80, 81).

In 2004, the last two genera within *Phycodnaviridae* were created to classify viruses infecting bloom-forming algae, each currently represented by a single species. The genus *Raphidovirus* includes viruses that infect and replicate in algae of the class Raphidophyceae, called *Heterosigma akashiwo* viruses (82). These viruses seem to establish a very specific relationship with their host, with some isolates capable of infecting only certain strains of *H. akashiwo* and generating viral progeny released through cell lysis (83, 84). It is noteworthy that *H. akashiwo* is also affected by RNA viruses (85), showing the remarkable viral diversity associated with algae, an area that remains poorly explored.

The sixth and final genus of *Phycodnaviridae* is *Coccolithovirus*, created to classify Emiliania huxleyi viruses (EhV), which infect the marine coccolithophore *Gephyrocapsa huxleyi* (also called *Emiliania huxleyi*) (86, 87). These algae exhibit a haplodiplontic life cycle, with the diploid form composed of a calcified layer (the coccolith) and susceptible to viral infection, while the haploid form appears to resist infection. This phenomenon was described as the “Cheshire Cat” strategy for escaping viral infection (88). Coccolithoviruses have some of the largest genomes among phycodnaviruses, exceeding the 400 kbp barrier (Fig. 3), and, unlike other members of the family, encode RNA polymerase homologs, giving them a more robust transcriptional machinery than other giant algal viruses (87). A comparative genomic analysis of 14 EhV isolates suggested a closed pan-genome for this group (89). However, because all isolates originated from geographically similar regions, future sampling from unexplored locations may reveal additional genomic diversity.

**Fig 4:**
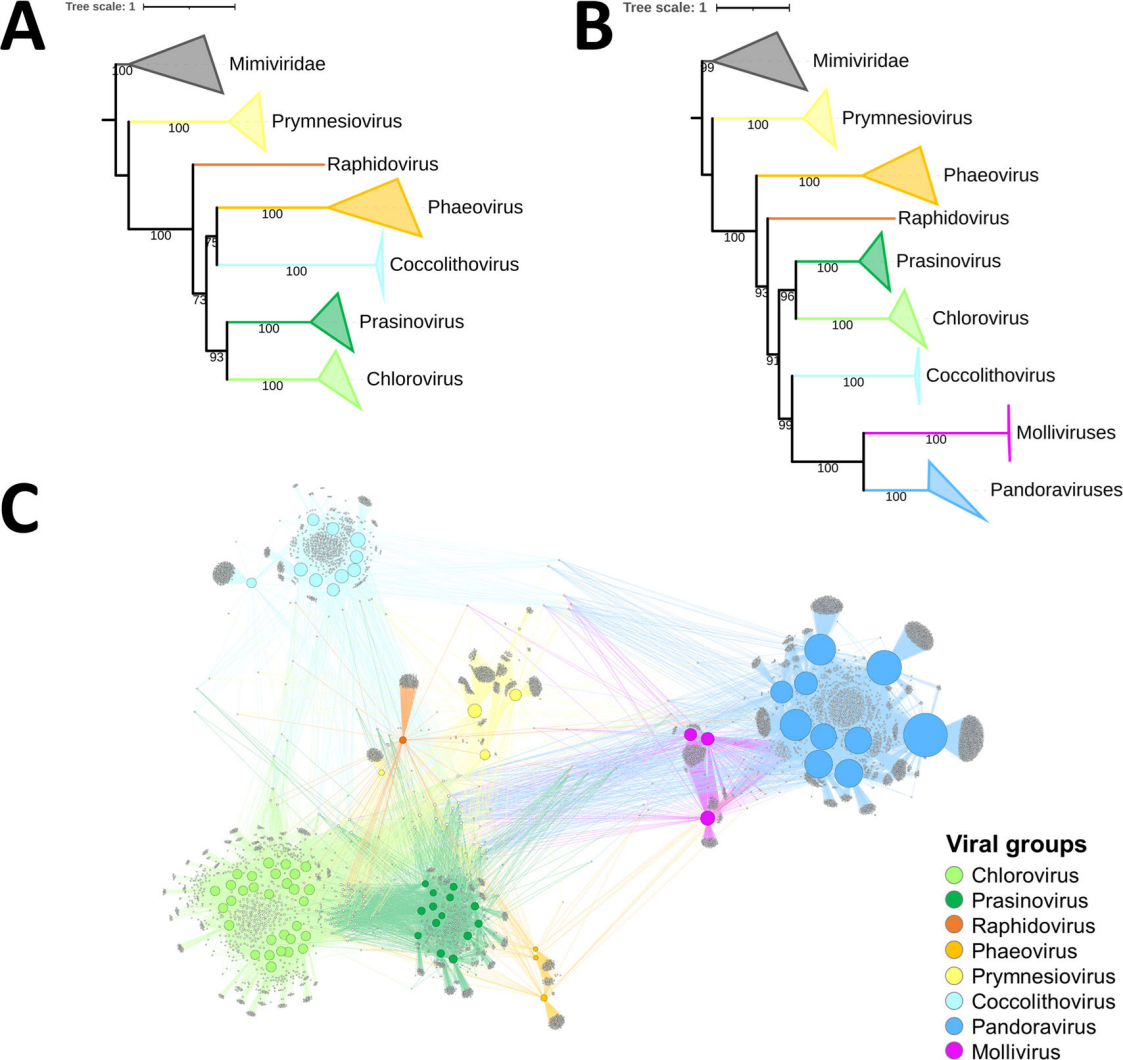
Phylogenetic relationship and COG-sharing pattern within *Algavirales*. (**A**) Maximum likelihood phylogenetic tree based on amino acid sequences of DNA polymerase B family, including isolated viruses from all genera of the family *Phycodnaviridae*, using viruses of the family *Mimiviridae* as the outgroup. (**B**) Same tree as in panel A, with data set supplemented with sequences from pandoraviruses and molliviruses, evidencing the phylogenetic proximity of these viruses with coccolithoviruses. Both trees were inferred using IQ-TREE2 with the LG + I + F + G4 evolutionary model and 1,000 bootstrap replicates (100), and visualized with iToL v6 (61). Scale bars indicate amino acid substitution rate. (**C**) Bipartite network graph showing the COG-sharing among phycodnaviruses, pandoraviruses, and molliviruses. COGs were constructed using ProteinOrtho with default parameters implemented in the Galaxy server (https://usegalaxy.fr/). CDSs from 76 viral genomes downloaded from GenBank were clustered, resulting in 8,221 COGs from a total of 35,476 genes, of which 4,463 were singletons. Colored nodes represent viruses from different viral groups according to the color key, and white nodes represent the COGs. Nodes’ sizes are related to the degree of connection. The graph was built using a force-based algorithm implemented in Gephi 0.10 (101), with minimal manual adjustment to allow clear visualization of the viral groups.

Since the creation of the family *Phycodnaviridae* in 1990, major advances have been made in understanding the diversity and complexity of giant algal viruses. Historically, these viruses were viewed as a monophyletic group, despite substantial differences in morphology, biology, and host range. Phylogenetic analyses using conserved genetic markers, such as the DNA polymerase gene, consistently place phycodnaviruses as a sister group to *Mimiviridae* (Fig. 4A), something observed in many previous studies (90–92). Almost 30 years later, in order to establish a viral megataxonomy, the ICTV created the taxon *Algavirales* in 2019, an order within the phylum *Nucleocytoviricota*, in reference to giant viruses that infect algae, whose only family included in the order at the time, and still today, is *Phycodnaviridae* (21). It is noteworthy that, when considering only isolated phycodnaviruses, *Algavirales* appears to form a monophyletic group within *Nucleocytoviricota* (Fig. 4A). However, this view was challenged by the discovery of two groups of giant viruses infecting free-living amoebae, the so-called pandoraviruses and molliviruses (93, 94). These viruses infect free-living amoebae of the genus *Acanthamoeba*, have round or oval particles up to 1 µm in size, and genomes that can reach 2.5 Mbp, as in the case of Pandoravirus salinum (93). Despite their distinct biological and morphological characteristics, phylogenetic analyses based on conserved genes place these viruses in close proximity to coccolithoviruses (Fig. 4B), which led to the initial proposition that they would represent highly derived phycodnaviruses (95). With the isolation and characterization of other molliviruses and pandoraviruses, the existence of new viral families with a phylogenetic relationship to coccolithoviruses becomes evident (96). Analyzing the pattern of COG sharing, it is also notable that coccolithoviruses exhibit a certain distance from other phycodnaviruses, especially chloroviruses and prasinoviruses, and share more COGs with amoeba viruses compared to other algal viruses (Fig. 4C). With this scenario, the monophyly originally conceived for *Algavirales* is disrupted, unless one considers that some amoeba viruses are also capable of infecting algae, something that has never been observed. Therefore, it is necessary to recognize that viral diversity based on viral isolation is quite limited and can lead to inaccurate interpretations of the evolutionary relationship between viruses, thus also affecting viral taxonomic classification. A major advance in this area has been the application of metagenomics to viral discovery and classification (97). The availability of new giant virus sequences derived from metagenomes (called GVMAGs) has revealed an unprecedented level of viral diversity (98), and the association of genomic information from isolated viruses with GVMAGs has begun to fundamentally reshape our understanding of giant virus taxonomy and evolution (48, 99).

## A NEW VISION FOR *ALGAVIRALES*

**Fig 5:**
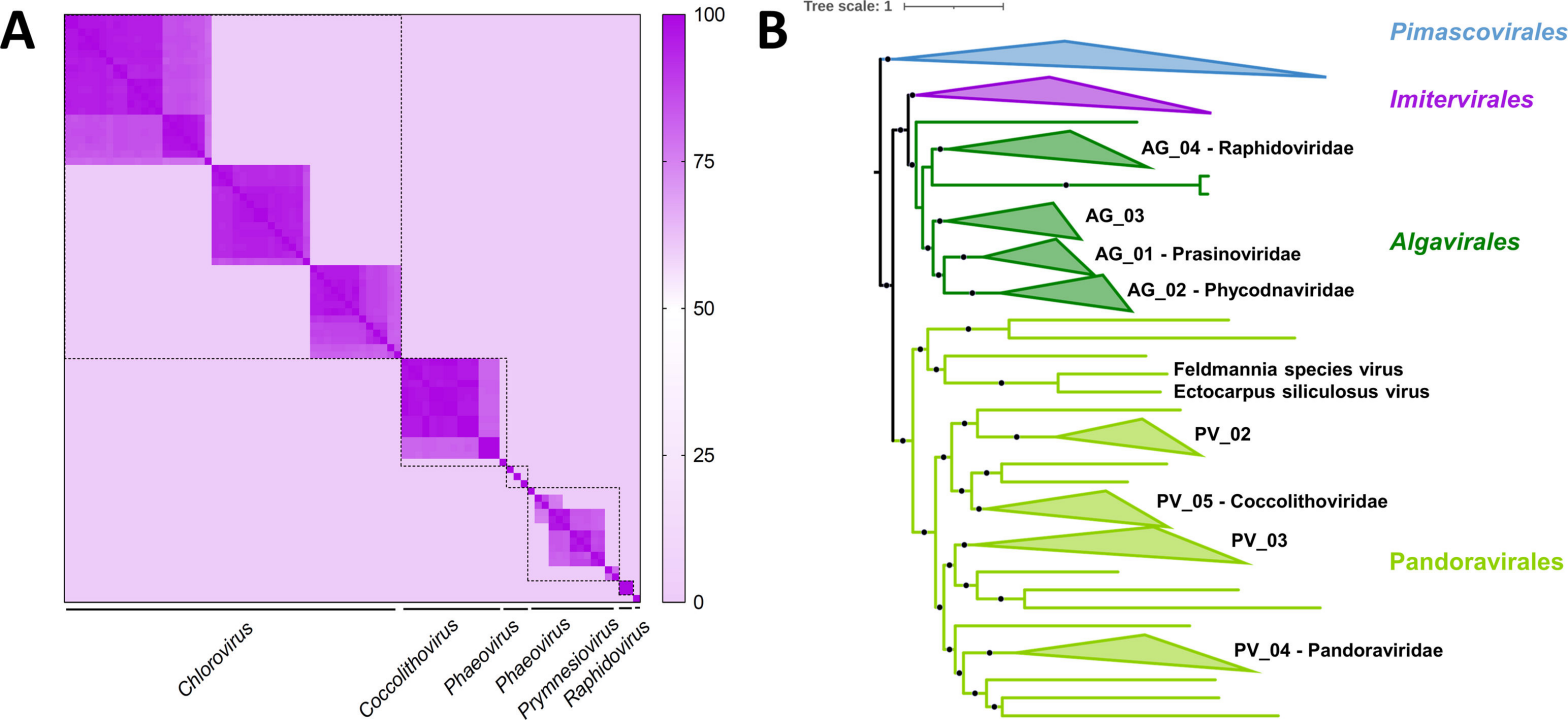
A putative new conformation of *Algavirales*. (**A**) Heatmap of ANI values comparing different viruses of the family *Phycodnaviridae*. ANI analysis was performed using FastANI implemented in Galaxy server (https://usegalaxy.fr/), including the genomes of 82 viruses across the six genera of *Phycodnaviridae*. Only ANI values above 75% were computed, and those below this threshold were set to zero for visualization. Dotted boxes indicate the virus genera. (**B**) Phylogenetic tree of the phylum *Nucleocytoviricota* evidencing the order *Algavirales* and the putative order “Pandoravirales,” with proposed families labeled. The tree was adapted from reference 98, previously published under a CC0 1.0 Universal license. Current genera and viruses of the family *Phycodnaviridae* are indicated in the tree, including those that correspond to putative families that may be considered in future taxonomic updates.

Taxonomy is a constantly evolving science. The proposal of a viral megataxonomy represented a milestone for virology, but the authors acknowledge that there are limitations and that changes are expected in light of new findings so that they better reflect the diversity and evolutionary history of viruses (21). The isolation and genomic characterization of more than 100 chloroviruses allowed us to advance the understanding of the diversity of these viruses and propose the creation of new taxonomic units, including 20 chlorovirus species (60). More importantly, it established criteria for organizing viruses into different hierarchical levels, in this case, subgenera and species. This framework can be extended to other genera within *Phycodnaviridae*. Applying an ANI threshold of 94% to available genomes of isolated phycodnaviruses suggests the existence of at least 14 subgenera and probably at least 35 viral species within the family (Fig. 5A). This is a preliminary analysis, and a more in-depth assessment and a more robust discussion should be made for the establishment of new taxa. However, there are different degrees of complexity when we think about taxonomic advances in virology. At this first moment, proposing additional species within the current structure of *Algavirales* is relatively straightforward, although limited by the small number of complete genomes available for most phycodnaviruses groups (Fig. 3). A far more complex challenge lies in reorganizing the order *Algavirales* in a manner that accurately reflects the biological, genomic, and evolutionary diversity of giant algal viruses. This is no reason, however, to avoid such a thought process. In fact, this discussion has already begun (99).

As previously mentioned, with the advancement of metagenomics, numerous GVMAGs have been identified, dramatically expanding our view of giant virus diversity (98). Only by incorporating these sequences can a robust phylogenetic reconstruction of giant viruses be achieved. Analyses based solely on isolated viruses inevitably rely on limited genetic information and risk producing incomplete or misleading taxonomic frameworks. It was using more than 1,300 giant virus sequences (1,253 GVMAGs and 130 isolated viruses) that a general phylogenetic framework of *Nucleocytoviricota* was established, based on a phylogenetic analysis with seven marker genes (99). Considering this data set, the maintenance of the order *Algavirales* and the creation of a new order “pandoravirales” were proposed. This new order would include pandoraviruses and molliviruses within the family “Pandoraviridae,” while coccolithoviruses would be reassigned from *Algavirales* to a new family, “Coccolithoviridae,” within pandoravirales, which would comprise at least four families (PV_02 to PV_05) (99) (Fig. 5B). It is worth noting that phaeoviruses would also be included within the new order “pandoravirales” (Fig. 5B). Under this scenario, *Algavirales* would encompass at least four families (AG_01 to AG_04). Prasinoviruses would be maintained but elevated to their own family, “Prasinoviridae,” whereas chloroviruses would remain within *Phycodnaviridae*, alongside two additional families, one of which would include raphidoviruses (Fig. 5B). Prymnesioviruses still need to be better evaluated and reference sequences included in the analyses. It is currently unclear whether they will remain within *Algavirales* or be reassigned to pandoravirales.

In this new scenario, the family *Phycodnaviridae* would be completely restructured, with all six genera elevated to family level. Aylward et al. proposed keeping chloroviruses within *Phycodnaviridae* (99). In their analysis, only 10 sequences were used, including six chlorovirus sequences. The other four were from GVMAGs obtained from freshwater (*n* = 2) and wastewater (*n* = 2). It is likely that additional chlorovirus-like viruses will be discovered in diverse environments, potentially infecting hosts beyond the Chlorellaceae family. Nevertheless, given the historical significance of the group, I also agree and support that the name “Phycodnaviridae” should be maintained, even if it initially encompasses only the genus *Chlorovirus*. With this, the restructured order *Algavirales* would continue to group giant DNA viruses that infect algae, as originally intended, but not all known giant algal viruses. With the discovery of many other giant algal viruses in recent years, it has become clear that the diversity of giant algal viruses is enormous and exceeds the limits of the current megataxonomy. This does not mean that what has been proposed should be abolished. Quite the contrary. A reorganization in light of the new data would be more reasonable, keeping in mind that this will probably not be the last time that taxonomic changes are made to giant algal viruses.

## CONCLUDING REMARKS

The discovery of chloroviruses was a milestone in virology, stimulating and culminating in the discovery of numerous other giant viruses, revealing a viral diversity and complexity previously unimaginable. The isolation and characterization of more than 100 chloroviruses demonstrated that these viruses exhibit remarkable genomic and biological diversity, enabling the proposal of robust criteria to advance their taxonomy (60). We hope that this work will be the starting point for new advances in order to improve the understanding of the diversity and evolution of giant algal viruses. To this end, isolating and characterizing new algal viruses will be essential to establishing a robust data set that serves as a reference for each viral group. This will allow advances not only in phylogenetic systematics but will also be the fundamental basis for advances in understanding the individuality of each virus. Chloroviruses that are nearly identical at the genomic level can display strikingly different biological traits (59), and it is likely that the same will happen for other giant algal viruses.

In an era dominated by omics-based approaches, it is crucial not to lose sight of the fact that classical virology remains equally, or even more, relevant, allowing for advances in understanding the biology of viruses beyond the molecular level. Only by integrating traditional virus isolation and experimental characterization with modern metagenomic analyses can we begin to appreciate the true extent of giant virus diversity and reconstruct their evolutionary history with confidence. We may never arrive at a definitive answer to the fundamental question posed at the beginning of this minireview. Nevertheless, the pursuit of this question has profoundly deepened our understanding of the virosphere in which we live. Thanks to the efforts and persistence of colleagues who have dedicated and continue to dedicate a good part of their careers to unraveling the mysteries of aquatic viruses, especially algal viruses, we now better appreciate the critical roles these viruses play in ecology, evolution, and biotechnology, among other fields. As Short et al. assertively wrote in a comprehensive review on algal viruses (12), “...knowing and understanding the viruses that infect algae is a vitally important scientific endeavor that reaches beyond the aquatic sciences.”
